# Shellfish Toxins Targeting Voltage-Gated Sodium Channels

**DOI:** 10.3390/md11124698

**Published:** 2013-11-28

**Authors:** Fan Zhang, Xunxun Xu, Tingting Li, Zhonghua Liu

**Affiliations:** Cooperative Innovation Center of Engineering and New Products for Developmental Biology, College of Life Sciences, Hunan Normal University, Changsha, Hunan 410081, China; E-Mails: zhangfan20111112@126.com (F.Z.); xxxu20120101@126.com (X.X.); Demi4869@126.com (T.L.)

**Keywords:** VGSCs, shellfish toxins, structure, bioactivity, pharmaceutical potential

## Abstract

Voltage-gated sodium channels (VGSCs) play a central role in the generation and propagation of action potentials in excitable neurons and other cells and are targeted by commonly used local anesthetics, antiarrhythmics, and anticonvulsants. They are also common targets of neurotoxins including shellfish toxins. Shellfish toxins are a variety of toxic secondary metabolites produced by prokaryotic cyanobacteria and eukaryotic dinoflagellates in both marine and fresh water systems, which can accumulate in marine animals via the food chain. Consumption of shellfish toxin-contaminated seafood may result in potentially fatal human shellfish poisoning. This article provides an overview of the structure, bioactivity, and pharmacology of shellfish toxins that act on VGSCs, along with a brief discussion on their pharmaceutical potential for pain management.

## 1. Overview of Voltage-Gated Sodium Channels

**Figure 1:**
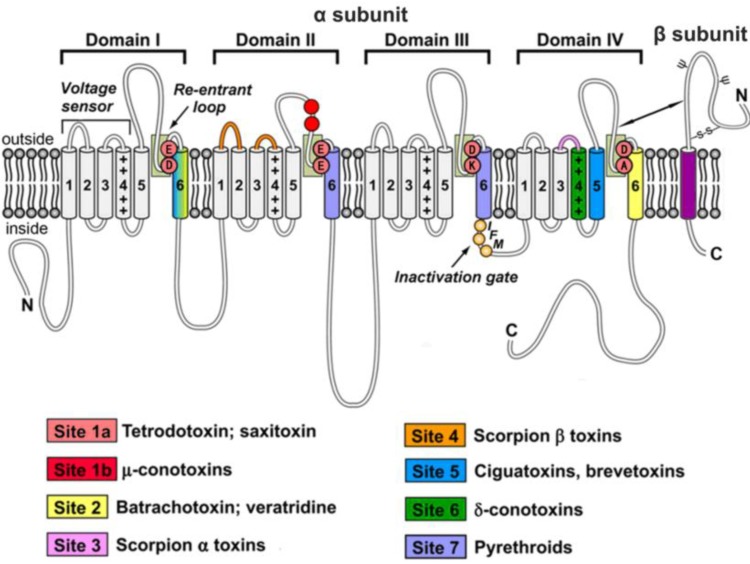
Schematic diagram of molecular structure and pharmacology of VGSCs. VGSCs comprise a core protein α-subunit and one or more auxiliary β-subunits. The α-subunit consists of four homologous domains designated I–IV. Each domain is comprised of six transmembrane helical segments (S1–S6), which are represented by cylinders. The central pore is formed by the transmembrane segments S5 and S6, ion selectivity filter is formed by the segments SS1 and SS2 (re-entrant loops, the light green box), and the voltage-sensor is formed by the transmembrane segments S1 to S4. The positively charged S4 segment is principally responsible for sensing changes in membrane potential, modulating channels to open or close. The fast inactivation gate is formed by intracellular linker between transmembrane domains III and IV and contains an IFM (orange balls), which plugs the pore and prevents Na^+^ internal flow. Auxiliary β-subunits of VGSCs are illustrated in red cylinders. N-linked carbohydrate chains are presented by ψ. Different colored regions represent seven neurotoxin receptor sites. Figure adapted from King *et al.* (2008, 2012) and Catterall *et al.* (2007) [9,10,11].

Voltage-gated sodium channels (VGSCs) are transmembrane proteins that form ion channels, conducting sodium ion (Na^+^) through the cell plasma membrane upon activation. VGSCs are responsible for the rising phase of action potentials in neurons, myocytes, and other excitable cells and play a key role in many electrophysiological processes [1,2]. VGSCs consist of an α-subunit (220–260 kDa) that forms the ion conduction core and one or more auxiliary β-subunits (30–40 kDa) [1,3,4,5,6,7]. The α-subunit consists of four homologous but non-identical transmembrane domains (I to IV), each of which contains six transmembrane segments (S1–S6) and a short membrane-penetrating segment (SS1 and SS2) between segments S5 and S6 (Figure 1) [1,8,9,10,11]. The α-subunit contains functional structures of the central pore (S5 and S6), ion selectivity filter (SS1 and SS2), and voltage sensors (S1 to S4) [8]. The S4 segment contains analogous and repeated motifs in which a lysine or arginine residue is followed by two hydrophobic amino acids [8,12]. Positive charges on the lysine or arginine residues, which are called gating charges, move across the electric field when membrane potential changes, resulting in conformational changes and opening of the central pore. The inactivation gate contains three hydrophobic amino acid residues Ile-Phe-Met (IFM) and is formed by an intracellular linker between transmembrane domains III and IV. Following movement of the voltage sensors, the inactivation gate plugs the pore and prevents further Na^+^ flow through the channel [13,14].

In mammalian cells, four auxiliary β-subunits (β_1_–β_4_; genes, *SCN1B*–*SCN4B*) have been identified up to now. The β-subunit consists of four domains including an extracellular *N*-terminal signal peptide, an immunoglobulin domain, a transmembrane domain, and an intracellular *C*-terminal domain [7]. Studies conducted on co-expression of α- and β-subunits have demonstrated that β-subunits modulate the kinetics and voltage-dependence of the opening and closing (gating) of the channel. More recently, β-subunits have also been reported to modulate cell adhesion and migration [7,15].

In mammalian cells, nine isoforms of VGSCs (Nav1.1–Nav1.9) have been identified and functionally expressed (Table 1) [2,16,17]. The nine isoforms are more than 50% identical in amino acid sequence in transmembrane and extracellular domains. Among the nine isoforms, Nav1.1–1.4, Nav1.6, and Nav1.7 are blocked by nanomolar concentrations of tetrodotoxin (TTX), a guanidinium toxin selectively targeting on VGSCs and generally being used as the agent to classify VGSCs, and, hence, categorized as TTX-sensitive (TTX-S) [18,19], while Nav1.5, Nav1.8, and Nav1.9 are resistant to micromolar or higher concentrations of TTX and are generally categorized as TTX-resistant (TTX-R). The nine VGSC isoforms have different tissue distributions [20]. Nav1.1–1.3 and Nav1.6 are mainly distributed in central nervous system (CNS), Nav1.7, Nav1.8, and Nav1.9 are highly expressed in peripheral nervous system (PNS), and Nav1.4 and 1.5 are selectively expressed in adult skeletal and heart muscles, respectively. Five isoforms of VGSCs, Nav1.1 and Nav1.6–1.9 are found to be expressed in dorsal root ganglion neurones. In addition to the nine isoforms, a potential tenth isoform (Nax) has been identified. It shows approximately 50% identity to the Nav1 subfamily of VGSCs. However, Nax exhibits key structural differences in functionally important regions of the voltage sensor and the inactivation gate. In addition, Nax expressed in heterologous cells is not functionally active, suggesting that Nax may not function as a VGSC.

A variety of toxins and drugs have been found to act on VGSCs. These compounds provide invaluable pharmacological tools for the investigation of VGSCs. The mechanisms of action of these agents have been studied by site-directed mutagenesis, binding studies and electrophysiological methods [1,9]. Based on their functional effects and receptor binding sites, neurotoxins targeting VGSCs are generally classified into three groups: (1) Pore-blocking toxins such as TTX, saxitoxin (STX), and μ-conotoxins, which block Na^+^ conductance via binding to the outer mouth of the pore (site 1); (2) toxins which bind to intramembrane receptor site 2 (e.g., veratridine, batrachotoxin, and grayanotoxin), or 5 (e.g., brevetoxin and ciguatoxin) in the activated state of channels, or site 7 (e.g., pyrethrins), resulting in persistent activation and negative shift in voltage-dependency of activation; (3) toxins which bind to extracellular receptor site 3 (e.g., α-scorpion toxins, sea-anemone toxins, and spider toxins) or 4 (e.g., β-scorpion toxins and spider toxins), resulting in delayed inactivation and shift in voltage-dependency of activation, respectively. Conotoxin-TxVIA binding to receptor site 6 produces the same physiological effects on Na^+^ currents as toxins binding to receptor site 3, though the binding of conotoxin-TxVIA is voltage-independent. Both, sites 6 and 3 are allosteric binding sites [1,21].

**Table 1:** Properties of the α-subunit of voltage-gated sodium channels (VGSCs) [2,16,17].

α-Subunits	Gene Symbol	Chromosomal Location1 ^♦^	TTX-S/R ^♦♦^	Predominant Location	Expression in DRG ^♦♦♦^	Effect of Mutation
Na_v_1.1	*SCN1A*	M:2 H:2q24	S	CNS, PNS	+++	Epilepsy
Na_v_1.2	*SCN2A*	M:2 H:2q23-24	S	CNS	+	Epilepsy
Na_v_1.3	*SCN3A*	M:2 H:2q24	S	CNS (embryonic)	upregulated after axotomy	None reported
Na_v_1.4	*SCN4A*	M:11 H:17q23-25	S	skeletal muscle	−	Myotonia, periodic paralysis
Na_v_1.5	*SCN5A*	M:9 H:3p21	R	heart muscle	−	Long-QT, Brugada syndrome, Progressive familial heart block
Na_v_1.6	*SCN8A*	M:15 H:12q13	S	CNS, PNS, glia nodes of Ranvier	+++	Cerebellar atrophy
Na_v_1.7	*SCN9A*	M:2 H:2q24	S	PNS Schwann cell	+++	Increased and decreased pain sensitivity
Na_v_1.8	*SCN10A*	M:9 H:3p22-24	R	PNS (sensory neurons)	+++	None reported
Na_v_1.9	*SCN11A*	M:9 H:3p21-24	R	PNS	+++	None reported
Na_x_	*SCN6A* (*SCN7A*)	M:2 H:2q21-23	R	heart, uterus, glia, PNS smooth muscle	+	−

♦ M: mouse, H: human. ♦♦ S: sensitive, R: resistant. ♦♦♦ +++: rich, +: present, −: absent. TTX: tetrodotoxin, DRG: dorsal root ganglion.

## 2. Shellfish Toxins

Shellfish toxins are a variety of potent toxic secondary metabolites primarily produced by prokaryotic cyanobacteria in both marine and fresh water ecosystems and eukaryotic dinoflagellates in marine water [22,23,24,25,26]. Many novel shellfish toxins have been reported in the last two decades [27,28,29]. In recent decades, dinoflagellates, cyanobacteria, and other algae have been causing harmful algal blooms (HABs) in many regions of the world with increasing frequency and global distribution owing to human activities and water eutrophication [30,31,32]. Some HAB species produce potent toxins, which are bioaccumulated in shellfish such as clams, oysters, whelks, mussels, conch, coquinas, and other filter-feeding molluscs via the food web [29]. Low concentrations of shellfish toxins usually produce no significantly adverse effects on shellfish, marine ecosystem, or humans. However, blooms of HAB species may result in accumulation of high concentrations of shellfish toxins in seafood, which could be fatally harmful to humans and cause a sharp downturn in seafood trade [29,30,31,32].

Shellfish toxins show diversity in structures and biological activities. Most shellfish toxins are neurotoxins which block nerve impulses via targeting specific receptors including voltage-gated Na^+^ , K^+^, and Ca^2+^ channels, specific nicotinic acetylcholine receptor (nAChR) subtypes, and Na^+^-K^+^ ATPase [33,34,35,36,37]. Consumption of shellfish toxin-contaminated seafood causes four major seafood-poisoning syndromes: paralytic shellfish poisoning, neurotoxic shellfish poisoning, amnesic shellfish poisoning, and diarrheic shellfish poisoning [27]. Since STX, the first and best-known member of paralytic shellfish toxins (PSTs), was discovered in 1957, 57 STX analogs have been reported to date [38,39]. STX, and analogs, selectively targeting VGSCs are valuable tool compounds for structure and function investigation of VGSCs [40,41]. More importantly, STX as a VGSC blocker prolongs the duration of anesthesia when combined with other local anesthetics [42]. Its analog gonyautoxin displays therapeutic potential in treatment of chronic anal fissure and chronic tension-type headache [43,44]. This review article provides an overview of chemistry, structure and bioactivity of shellfish toxins acting on VGSCs. Both widely known toxins (e.g., saxitoxins, gonyuantoxins, and brevetoxins) and recently discovered toxins (e.g., crossbyanols, hoiamide A and B, and Palmyramide A) are reviewed. We also provide a brief discussion on the pharmaceutical potential of shellfish toxins.

### 2.1. STX

STX and its analogs are highly potent neurotoxins known as PSTs. PSTs are produced by marine dinoflagellates and several fresh water species of cyanobacteria [22,23,24,27]. In the marine ecology, three species of dinoflagellate produce STXs, belonging to the genera *Alexandrium*, *Pyrodinium* and *Gymnodinium*, while five filamentous species of cyanobacteria in freshwater environment, including *Anabaena circinalis*, *Aphanizomenon* sp., *Aphanizomenon gracile*, *Cylindrospermopsis raciborskii*, and *Lyngbya wollei*, have been reported to produce STXs. Blooms of these toxic species have generated lethal impact on marine and freshwater ecosystems and humans, causing paralytic shellfish poisoning in humans, mass deaths of fish, birds, and other native animals and livestock, and contamination of freshwater resources [45,46,47,48,49].

Among marine neurotoxins, STX is one of the most toxic and the most harmful to humans, which usually causes paralytic shellfish poisoning (PSP). Inadvertent ingestion of STX or its analogs quickly causes symptoms of poisoning within 30 min, including a tingling or burning sensation in the lips, tongue, and throat, and facial numbness. Additional symptoms may include perspiration, diarrhea, and vomiting [50,51,52]. In cases of acute and severe poisoning, numbness may quickly spread to the neck and extremities, and symptoms may further develop to muscular weakness, loss of motor coordination, and, eventually, paralysis. Serious neurological symptoms may culminate in respiratory arrest, cardiovascular shock, and death [53]. The lethal oral dosage in human is 1–4 mg/person, depending on the physiological condition and gender of the victim [27,51]. There are currently no clinically approved antidotes to STX poisoning. Common treatments during early stages of PSP include removal of unabsorbed toxins with activated charcoal and artificial respiration. The half-life of STX in human is approximately 90 min. Survival opportunity significantly increases after 12 h from initial exposure [52,54]. STX and its analogs differ significantly in toxicity, with *N*-sulfo-carbamoyl derivatives being 10- to 100-fold less potent than the carbamate analogs [51,55]. STX and its analogs may potentially be used as biochemical weapons and have been listed as Schedule 1 chemical intoxicants by the Organization for the Prohibition of Chemical Weapons (OPCW). Their manufacture, use, transfers, and reuses are hence strictly regulated by OPCW [27].

**Figure 2:**
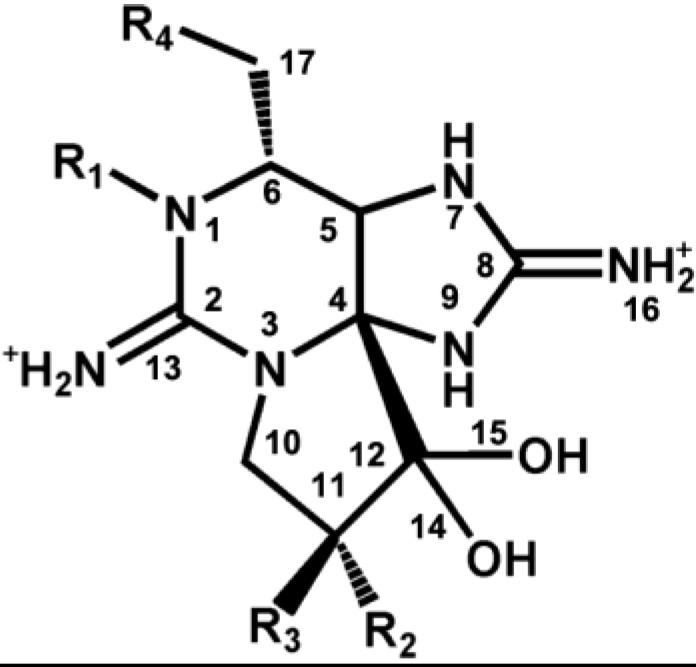
Structures and species of STX, and its analogs, produced by marine dinoflagellates. Figure adapted from Wang (2008) [27,62].

STX was first isolated in pure form from Alaskan butter clam, *Saxidomus gigangteus* in 1957 [39]. However, its crystal structure was not determined until 1975 due to the difficulty in finding the appropriate crystallization condition [56,57]. STX (C_10_H_17_N_7_O_4_, MW: 299 Da) is a heat-stable and water-soluble neurotoxic alkaloid. The core structure of STX and its analogs is a trialkyl tetrahydropurine, with the NH_2_ groups at positions 2 and 8 forming the two permanent guanidinium moieties [57,58,59,60]. STX has two pKa values, 8.22 and 11.28, for the 1, 2, 3 and 7, 8, 9 guanidinium groups, respectively [58,61]. STX and its analogs can be divided into four structural categories based on variations in functional groups at four defined positions (Figure 2): (1) Carbamate toxins containing a carbamoyl group at the R1 position, such as STX, neo-saxitoxin, and gonyautoxins 1–6; (2) *N*-sulfocarbamoyl toxins, such as C1–C4; (3) decarbamoyl toxins; and (4) deoxydecarbamoyl toxins [27,62].

**Table:** 

	Toxin	R1	R2	R3	R4
Carbamate	STX	H	H	H	OCONH_2_
	Neo STX	OH	H	H	OCONH_2_
	GTX1	OH	OSO_3_^−^	H	OCONH_2_
	GTX2	H	OSO_3_^−^	H	OCONH_2_
	GTX3	H	H	OSO_3_^−^	OCONH_2_
	GTX4	OH	H	OSO_3_^−^	OCONH_2_
*N*-sulfocarbamoyl	GTX5(B1)	H	H	H	OCONHSO_3_^−^
	GTX6(B2)	OH	H	H	OCONHSO_3_^−^
	C1	H	OSO_3_^−^	H	OCONHSO_3_^−^
	C2	H	H	OSO_3_^−^	OCONHSO_3_^−^
	C3	OH	OSO_3_^−^	H	OCONHSO_3_^−^
	C4	OH	H	OSO_3_^−^	OCONHSO_3_^−^
Decarbamoyl	dcSTX	H	H	H	OH
	dcNeoSTX	OH	H	H	OH
	dcGTX1	OH	OSO_3_^−^	H	OH
	dcGTX2	H	OSO_3_^−^	H	OH
	dcGTX3	H	H	OSO_3_^−^	OH
	dcGTX4	OH	H	OSO_3_^−^	OH
Deoxydecarbamoyl	doSTX	H	H	H	H
	doGTX2	H	H	OSO_3_^−^	H
	doGTX3	H	OSO_3_^−^	H	H

STX was the first discovered and the most well known potent neurotoxin that selectively targets VGSCs besides TTX. STX binds to VGSCs with high affinity (Kd ~2 nM), resulting in blockade of muscle action potential and respiratory paralysis [27]. Three models have been proposed for the mechanisms of VGSC blockage by STX [27]. Hille *et al.* postulated a plugging model in 1975, in which STX penetrates the VGSC protein and binds at the bottom of the channel, forming an ion pair with an anionic site rather than deeply plugging the channel. Nonetheless, the plugging model is unsuitable for unfolded gonyautoxins owing to the lack of anticipated steric interactions [63]. Subsequently, Kao and Walker proposed another model in which the toxin molecule binds at the outside edge of the channel with the guanidinium group on top of the channel entrance [64]. Meanwhile, Shimizu proposed a third model called three-point binding model, in which two hydrogen bonds with the ketal OHs and ion pairing of the guanidinium group with an anionic site on the outside surface of the membrane are speculated [65]. However, all the three models did not explain the precise action mechanism of the blockage of VGSCs by STX on molecular level until the successful cloning of VGSCs and the subsequent identification of the binding site of STX in the channel.

In 1986, Numa and Noda reported the first successful cloning of VGSCs [66]. Later, Numa and coworkers pioneered using site-directed mutagenesis to investigate the binding site of STX in Nav1.2. Their results demonstrated that changing glutamic acid residue 387 to glutamine (E387Q) and mutations of charged amino acid residues of SS2 in the four domains strongly reduced binding sensitivity. STX was found to bind to Nav1.2 through electrostatic interactions between the 7, 8, 9-guanidinium moiety of STX and certain fixed amino acid residues in the lip of the channel pore [64,67]. These fixed amino acid residues are located in segment SS2 of the S6 transmembrane segment in all four domains, forming two rings that interact with the STX molecule [40,41]. The first ring contains D384 in domain I, E942 in domain II, K1423 in domain III, and A1714 in domain IV. The second ring contains E387 in domain I, E945 in domain II, M1425 in domain III, and D1717 in domain IV. Therefore, studies reported thus far demonstrate that STX blocks VGSCs through binding to receptor site 1 [40,41]. The specific binding mode enables STX to effectively block the inward flow of Na^+^ into cell. More recently, Walker *et al.* reported a two amino acid variation of human Nav1.7, which results in dramatic differences in the binding affinity for STX; the two residues are Thr1398 and Ile1399 of domain III, which occur as Met and Asp in other VGSC isoforms, as the critical determinants of STX binding affinity. Their findings provide possibility that selective blockers of hNav1.7 could be designed around the site 1 for the uniqueness of its outer pore structure [68].

STX also binds to voltage-gated K^+^ and Ca^2+^ channels, however, the binding mode is different from that with VGSCs. STX modulates the gating of human K^+^ channels rather than directly blocking the pore through binding to extracellular sites of the channel [34]. For Ca^2+^ channels, STX was found to possibly bind to at sites located in the selectivity filter, resulting in incomplete blockage of the channel [35].

### 2.2. Brevetoxins

**Figure 3:**
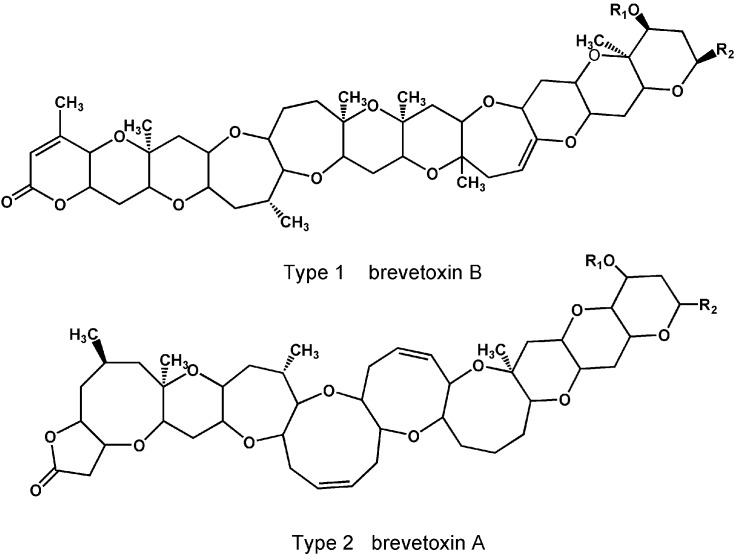
Structures of brevetoxins from marine dinoflagellates [27,62].

In marine ecosystems, dinoflagellates produce two types of lipid soluble toxins that cause neurotoxic shellfish poisoning with lethal effects on marine life including fish, sea birds, and marine mammals [49,69,70,71,72,73,74,75]. Brevetoxins are known as neurotoxic shellfish toxins, and they are mainly produced by dinoflagellate *Kerenia brevis*. However, brevetoxins were also recently found in other genera: *Chatonella marina* such as *C. antiqua*, *Fibrocapsa japonica*, *Heterosigma akashiwo*, *K. mikimotoi*, *K. brevisulcata*, *K. selliformis*, and *K. papilionacea* [27,29,69,70,76,77,78]. Brevetoxins are a suite of ladder-like cyclic polyether compounds with two types of backbone structures, brevetoxin B backbone (type 1: PbTx-2, 3, 5, 6, 8, and 9) and brevetoxin A backbone (type 2: PbTx-1, 7, and 10) (Figure 3) [27,62].

**Table:** 

Toxin	Type	R1	R2	Nominal Mass
PbTx-1	2	H	CH_2_C(CH_2_)CHO	866
PbTx-2	1	H	CH_2_C(CH_2_)CHO	894
PbTx-3	1	H	CH_2_C(CH_2_)CH_2_OH	896
PbTx-5	1	COCH_3_	K-ring acetate PbTx-2	936
PbTx-6	1	H	K-ring epoxide PbTx-2	910
PbTx-7	2	H	CH_2_C(CH_2_)CH_2_OH	868
PbTx-8	1	H	CH_2_COCH_2_Cl	916
PbTx-9	1	H	CH_2_CH(CH_3_)CH_2_OH	898
PbTx-10	2	H	CH_2_CH(CH_3_)CH_2_OH	870

The major symptoms of severe brevetoxin poisoning are paresthesia, nausea, abdominal pain, vertigo, ataxia, diarrhea, burning pain in the rectum, headache, and bradycardia. Brevetoxins may also cause respiratory irritation syndrome [79]. Brevetoxins are odorless, tasteless, and heat and acid stable, and, hence, not easily detectable in human food sources [27,29]. Of the ten brevetoxins identified so far, PbTx-3 was investigated more intensively compared with other ones. The LD_50_ of PbTx-3 in mice is 94 µg/kg body weight by intravenous injection, 170 µg/kg body weight by intraperitoneal injection, and 520 µg/kg body weight by oral administration, respectively [27,80]. Pathogenic dose of PbTx-3 in human is about 42–72 times larger than that in mice [27,80].

Brevetoxins inhibit the inactivation of TTX-S VGSCs through binding to site 5. Earlier investigations showed that brevetoxins activate VGSCs and lead to uncontrolled Na^+^ influx into the cell [70]. Subsequent studies by Poli and co-workers using [3H]brevetoxin PbTx-3 as a specific probe revealed that PbTx-3 does not bind to any of the previously described sites and a new site of binding, site 5, was identified [72]. Later studies further confirmed that brevetoxins are site 5 toxins, interacting with the α-subunit of VGSCs in a “head-down” orientation in a 1:1 stoichiometry. Binding of brevetoxins to site 5 causes conformational changes in the channel, which lead to abnormal opening of the channel and inhibited channel inactivation, resulting in persistent channel activation, membrane depolarization, Na^+^ current increment, and repetitive firing [27,81,82,83,84].

### 2.3. Antillatoxins

**Figure 4:**
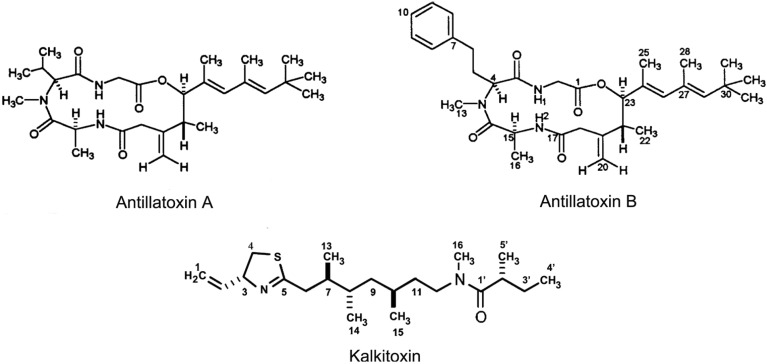
Structures of antillatoxin A, antillatoxin B and kalkitoxin [85,89,90].

Antillatoxin A (C_28_H_45_N_3_O_5_; [M + H]^+^ at *m/z* 504.3436) (Figure 4), a potent ichthyotoxin and cytotoxin, was first isolated from tropical marine cyanobacterium *Lyngbya majuscula* in Curaçao by Gerwick *et al.* [85]. It is a structurally unique cyclic lipopeptide composed of a tripeptide linked to a highly methylated lipid moiety via ester and amide bonds [85,86,87]. Antillatoxin A has been demonstrated to be one of the most ichthyotoxic metabolites isolated from marine algae up to now [85]. Blooms of *Lyngbya majuscula* have been associated with adverse effects on human health, including respiratory irritation, eye inflammation and severe contact dermatitis in exposed individuals [88].

The earliest report about antillatoxin A provided information about its basic structure features including seven partial structures by 1D- and 2D-^1^H and ^13^C NMR [85,91]. Yokokawa *et al.* reported the first successful total synthesis of (4*S*, 5*R*)-antillatoxin A and presented a proposed structure of antillatoxin A [92]. Later, Li *et al.* synthesized four different antillatoxin stereoisomers which are all likely C-4 and C-5 diastereomers, and identified possible stereochemistry of natural antillatoxin A through the study of the biological activities of the compound [87]. Total synthesis of antillatoxin A and its stereoisomers were explored and finally accomplished by Yokokawa and Shioiri (1998), Yokokawa *et al.* (1999, 2000), and Lee and Loh [86,92,93].

Antillatoxin A is one of the most ichthyotoxic metabolites as evaluated in the goldfish toxicity assay (LD_50_, 0.05 μg/mL; ED_50_, approximately 0.01 μg/mL) [85]. Berman *et al.* found that antillatoxin A showed concentration-dependent cytotoxicity to rat cerebellar granule cells with an LC_50_ value of 20.1 ± 6.4 nM [94]. Antillatoxin A has been demonstrated to be an activator of VGSCs, but its precise binding site on VGSCs remains unknown [95,96,97,98]. Li and co-authors reported that the rapid increase in intracellular Ca^2+^ produced by antillatoxin A was antagonized by TTX; Antillatoxin A allosterically enhanced the activity of [3H]batrachotoxin ([3H]BTX), a neurotoxin acting on site 2 of VGSCs; meanwhile, antillatoxin A in combination with brevetoxin produced a strong synergistic stimulation of [3H]BTX; furthermore, antillatoxin A enhanced Na^+^ influx in cerebellar granule cells by acting on VGSCs [95]. Subsequently, Cao *et al.* reported that high concentrations of antillatoxin A increased intracellular Na^+^ concentration, and this effect was abrogated by TTX [96]. Recently, the study from the same group proved that antillatoxin A was able to promote Na^+^ influx in cells heterologously expressing rat Nav1.2, rat Nav1.4, or rat Nav1.5 α-subunits by using the Na^+^ selective fluorescent dye, sodium-binding benzofuran isophthalate. They also found that potency of antillatoxin A on the three VGSC isoforms did not differ significantly and its efficacy was quite different from those of other VGSCs activators acting on sites 2 or 5 [98]. Taken together, these data demonstrate that antillatoxin A is an activator of VGSCs with unique pharmacological properties; decoding the molecular determinants and mechanism of action of antillatoxin A may provide further insight into gating mechanisms of VGSCs [98].

Antillatoxin B (C_33_H_48_N_3_O_5_ ; [M + H]^+^ at *m/z* 566.3596) (Figure 4), first isolated by Nogle *et al.* in 2001 from *Lyngbya majuscule* collected from Puerto Rico and the Dry Tortugas, is an *N*-methyl homophenylalanine homolog of antillatoxin A [89]. Similar to antillatoxin A, antillatoxin B activates VGSC expressed in mouse neuro-2a neuroblastoma cells (EC_50_ 1.77 μM) and shows strong ichthyotoxic activity (EC_50_ 1 μM). Antillatoxin B, however, is 10-fold less potent than antillatoxin A, indicating that the substitution at the *N*-methyl group is critical for compound potency [89].

### 2.4. Kalkitoxin

Kalkitoxin (C_21_H_38_N_2_OS; [M + H]^+^ at *m/z* 366.2696) (Figure 4), a thiazoline ring-containing lipopeptide, was first isolated by Wu in 1996 from organic extracts of *Lyngbya majuscula* collected in coasts of Curaçao using a brine shrimp toxicity guided assay. Later, Wu and coworkers reported its structure, synthesis, and biological properties [90]. In 2003, Nogle and Gerwick also reported the isolation of this potent neurotoxin from *Lyngbya majuscula* specimens collected in shallow waters off the coast of Puerto Rico [99]. Kalkitoxin is a valuable target for total synthesis for its interesting biological activities, intriguing structure, and scarcity in natural products. Wu *et al.* and White *et al.* successfully completed the total synthesis of (+)-kalkitoxin [90,100,101]. Recently, Umezawa and co-workers successfully synthesized kalkitoxin and its analogs and tested their biological activities using brine shrimp toxicity assay [102].

Natural kalkitoxin consists of a lipophilic chain, a 2, 4-disubstituted thiazoline, and an unsaturated CH_2_=CH_2_ unit [90]. There are five asymmetric centers in kalkitoxin structure including a thiazoline ring and four methyl groups. The absolute stereochemistry of natural kalkitoxin was determined to be 3*R*, 7*R*, 8*S*, 10*S*, and 2′*R* by comparing the ^13^C spectrum of the natural metabolite with those of synthesized compounds with all possible configurations. In 2004, White and coworkers published the full details of their work, which was a prerequisite for successful total synthesis of natural (+)-kalkitoxin [101].

In 2000, Wu *et al.* reported that natural (+)-kalkitoxin was strongly toxic to common goldfish (*Carassius auratus*, LC_50_ 700 nM) and brine shrimp (*Artemia salina*, LC_50_ 170 nM) [90]. Kalkitoxin also displayed extremely potent cytotoxicity (LC_50_ 3.86 nM) to primary cultures of rat neurons, which was antagonized by NMDA receptor antagonists dextrorphan and MK-801 [94]. (+)-Kalkitoxin and two synthetic precursors showed cytotoxicity to human colon cell line HCT-116, indicating that the thiazoline moiety of kalkitoxin is required for cytotoxicity [101].

Natural (+)-kalkitoxin is an antagonist of VGSCs. However, there have been few reports about its action mechanisms and binding site on VGSCs. Wu *et al.* demonstrated that kalkitoxin was a very potent blocker of VGSCs expressed in mouse neuro-2a cells (EC_50_ of kalkitoxin, 1 nM *vs.* EC_50_ of saxitoxin, 8 nM) [90]. In 2005, LePage *et al.* found that kalkitoxin interacted with VGSCs in cerebellar granule neurons (CGN) [103] and eliminated elevation of [Ca^2+^]_i_ induced by veratridine (a VGSCs activator) and neurotoxicity in CGN in a concentration-dependent manner (EC_50_ 262.7 nM), providing indirect evidence that kalkitoxin may be a blocker of TTX-S VGSCs. Kalkitoxin showed no effect on basal binding of [3H]BTX to VGSCs, but it inhibited [3H]BTX binding to VGSCs in the presence of deltamethrin, a positive allosteric modulator of VGSCs. These results also suggest that kalkitoxin may be a blocker of VGSCs for that BTX binds to VGSCs only when the channel is in the open conformation [91,103].

### 2.5. Jamaicamides

**Figure 5:**
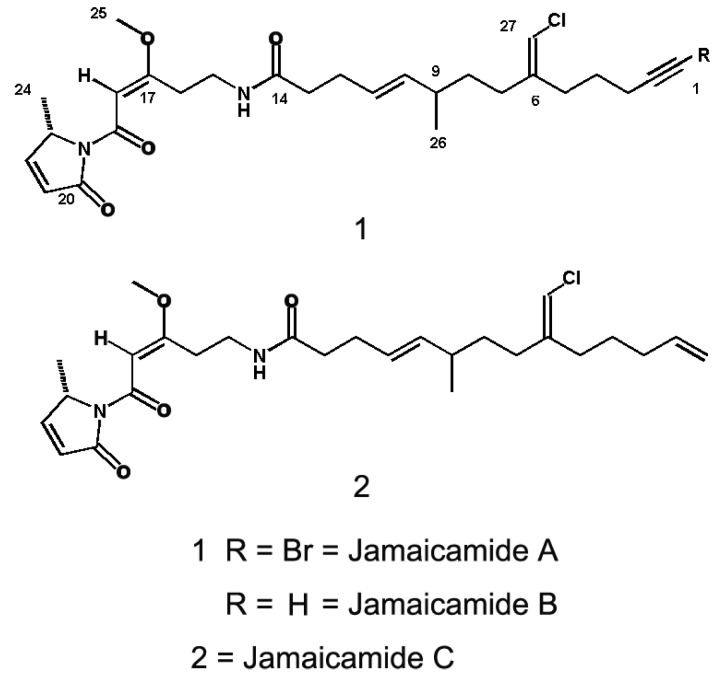
Structures of Jamaicamide A–C [104].

Jamaicamides include jamaicamide A, B, and C. Jamaicamide A (C_27_H_37_O_4_N_2_ClBr; [M + H]^+^ at *m/z* 567.1625) (Figure 5) is a novel and highly functionalized lipopeptide, containing an alkynyl bromide, a vinyl chloride, a β-methoxy eneone system, and a pyrrolinone ring [104]. It was first isolated from a dark green strain of *Lyngbya majuscula* (strain JHB) by Edwards *et al.* in 2004. Jamaicamide B (C_27_H_37_O_4_N_2_Cl) was first isolated as a pale yellow oil from the lipid extract of cultured *Lyngby majuscule* JHB and is slightly more polar than jamaicamide A. Jamaicamide C (C_27_H_39_O_4_N_2_Cl) was purified from the crude extract of cultured *Lyngby majuscule* JHB in very low yield (0.5%), and is slightly more hydrophobic than either jamaicamide A or B.

The high-resolution fast atom bombardment mass spectrometry and isotope peak analyses of the structure of jamaicamide A highlighted the presence of a chlorine atom and a bromine atom in the molecule [104]. Its degree of unsaturation was calculated to be 10 from its molecular formula [104]. Similarly, jamaicamide B also has a degree of unsaturation of 10, with ^1^H and ^13^C resonances very close to those of jamaicamide A. The only difference is the lack of bromine signal in jamaicamide B, suggesting that jamaicamide B is the debromo analog of jamaicamide A. The molecular formula of jamaicamide C was determined to be C_27_H_39_O_4_N_2_Cl by the high-resolution fast atom bombardment mass spectrometry with a deduced degree of unsaturation of 9. ^1^H and ^13^C NMR data indicate that jamaicamide C has two additional hydrogen atoms compared with jamaicamide B. Recently, Graf *et al.* published the total synthesis of jamaicamide A [105].

Jamaicamides have potent ichthyotoxicity and cytotoxicity [104]. In a goldfish toxicity assay, jamaicamide B was the most active (100% lethality at 10.22 nmol after 90 min) followed by jamaicamide C (100% lethality at 20.36 nmol after 90 min), while jamaicamide A was the least active fish toxin (sublethal toxicity at 17.62 nmol after 90 min). In a brine shrimp toxicity assay, jamaicamide C was only modestly active (20.36 nmol, 25% lethality) while both jamaicamide A and B showed no significant toxicity. Moreover, jamaicamides A, B, and C exhibited similar cytotoxicity to both H-460 human lung cell line and Neuro-2a mouse neuroblastoma cell line (LC_50_ ~15 μM).

Jamaicamides are also antagonists of VGSCs. Edwards and coworkers reported that jamaicamides A, B, and C at 5 μM all partially blocked VGSC activity with percent inhibition about half of that by saxitoxin at 0.15 μM using an antagonism cell bioassay [104]. In this assay, the ability of jamaicamides to antagonize the combined cytotoxic effects of ouabain (a plasma membrane Na^+^-K^+^ ATPase inhibitor) and veratridine on cerebellar granule neurons were examined, and the results were scored through visually noting morphology of a significant number of living cells [106].

### 2.6. Crossbyanols

**Figure 6:**
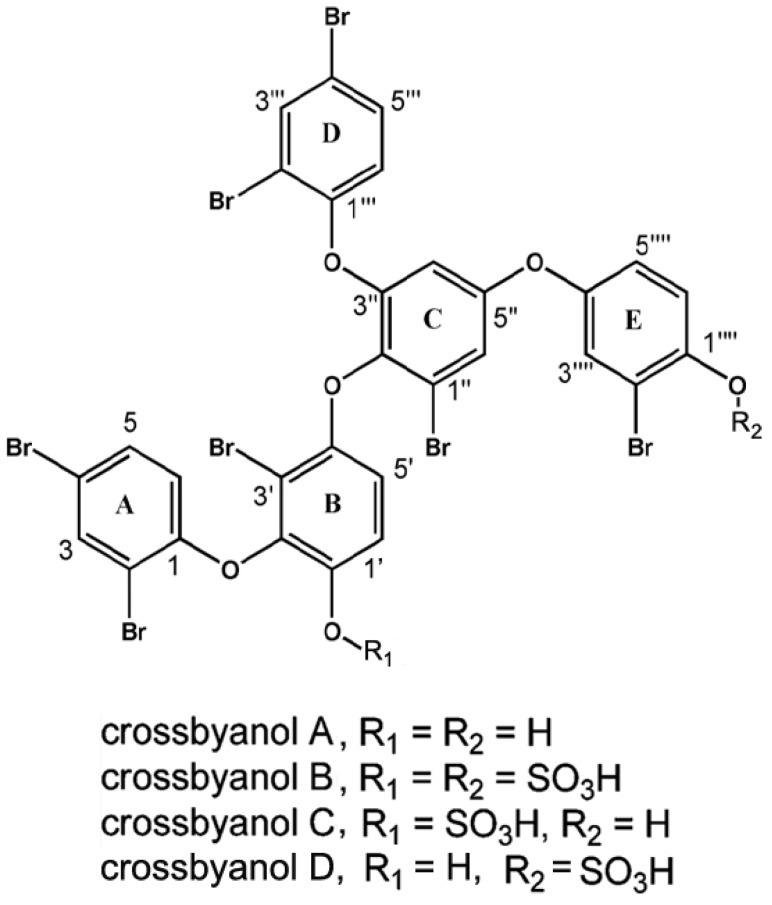
Structures of crossbyanol A–D [107].

Crossbyanols A–D are four heptabrominated polyphenolic ethers first isolated from marine cyanobacterium *Leptolyngbya crossbyana* collected at Honaunau reef off the island of Hawaii [107]. The periodic extensive blooms of *Leptolyngbya crossbyana* in Hawaii coral reefs cause significant damage to the subtending corals, generating serious concerns. Chemical investigation of bioactive secondary metabolites of *Leptolyngbya crossbyana* led to the discovery of crossbyanols. Crossbyanols have a branched oligomeric structure of polybrominated diphenyl ethers [107]. Crossbyanol A (C_30_H_15_^79^Br_7_O_6_, *m/z*: 1046.5044) possesses seven bromine atoms, three 1,2,4-trisubstituted phenyl rings, one 1,2,3,4-tetrasubstituted phenyl ring, and one 1,2,3,5-tetrasubstituted phenyl ring (Figure 6). Crossbyanol B (C_30_H_15_^79^Br_7_O_12_S_2_, *m/z*: 1182.4172) shows an isotope pattern similar to that of crossbyanol A with the presence of two additional sulfate groups. Interestingly, crossbyanol C and crossbyanol D showed identical molecular ion peaks at *m/z* values of 1102.4703 and 1102.4642 in negative-ion HRESIMS, suggesting that they share identical molecular formula of C_30_H_15_^79^Br_7_O_9_S. Similarly, crossbyanol C and D contain the same five substructures with substitution patterns identical to that of crossbyanol A or B. In addition, crossbyanol C and D have one sulfate group and one hydroxyl group in their structures [107].

Among crossbyanols, crossbyanol A activates VGSCs expressed in neuro-2a mouse neuroblastoma cells, while crossbyanols B, C, and D do not [107]. However, how crossbyanol A activates VGSCs is largely unknown. Most studies on crossbyanols A–D are mainly about their antibacterial, ichthyotoxic, and cytotoxic activities [107]. Crossbyanol A has weak cytotoxicity against H-460 human lung cancer cells (IC_50_ 30 µg/mL). Crossbyanol B displays the most potent antibacterial activity (MIC value: 2.0–3.9 µg/mL) against methicillin-resistant *Staphylococcus aureus*. In addition, crossbyanol B is potently ichthyotoxic to brine shrimp with an IC_50_ value of 2.8 µg/mL. However, crossbyanols C and D show no bioactivity in all these assays at the maximum test concentration of 20 µg/mL. The observation that crossbyanols A and B have quite different bioactivities in these assays suggests that the sulfation of crossbyanols is critical for potent biological activities in this structure class. It is not clear if VGSC stimulation activity of crossbyanol A correlates with its cytotoxicity [107].

### 2.7. Hoiamides

**Figure 7:**
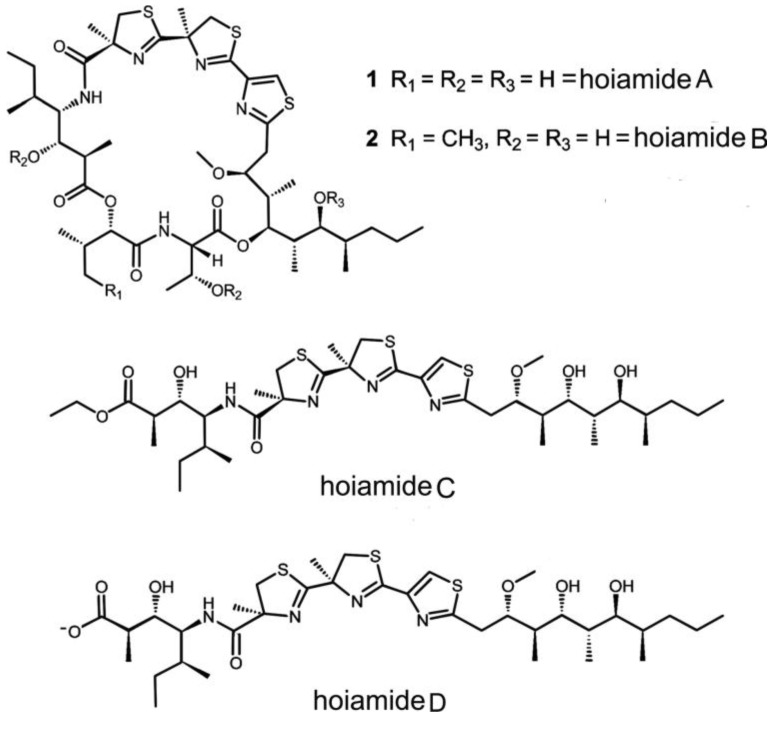
Structures of hoiamide A–D [108,109,110].

Hoiamides A–D are a new family of marine secondary metabolites. In 2009, the first member of hoiamides, hiamides A (C_44_H_71_N_5_O_10_S_3_, *m/z*: 926.4441) (Figure 7), was isolated from marine cyanobacteria *Lyngbya majuscula* and *Phormidium gracile* collected in Papua New Guinea [108]. Hoiamide A, a novel cyclic depsipeptide of a highly unusual structure, displays potent cytotoxicity and neurotoxicity [108]. In 2010, Gerwick *et al.* reported two additional compounds in the hoiamide family, the cyclic depsipeptide hoiamide B (C_45_H_73_N_5_O_10_S_3_, *m/z*: 940.4584) and the linear lipopeptide hoiamide C (C_37_H_62_N_4_O_7_S_3_, *m/z*: 770.3775), which were purified from two different collections of marine cyanobacteria obtained in Papua New Guinea, respectively [109]. The fourth member, hoiamide D (C_35_H_58_N_4_O_7_S_3_, *m*/*z*: 743.3535) was isolated from cyanobacterium *Symploca* sp. collected in Kape Point, Papua New Guinea [110].

The hoiamides A–D are a new class of cyanobacterial compounds featuring a triheterocylic system likely derived from a mixed peptide-polyketide biogenetic origin. The hoiamides are composed of a peptidic segment with an acetated and *S*-adenosyl methionine modified isoleucine moiety, a central triheterocyclic core comprised of two α-methylated thiazolines and one thiazole, and a highly oxygenated and methylated C-15 polyketide substructure [108,109]. Particularly, hoiamide A and B are cyclic while hoiamide C and D are linear. Moreover, hoiamide C may be an extraction byproduct of hoiamide D given the utilization of ethanol in the isolation and storage of the biological material [109]. The unique structures and bioactivities of hoiamides elicited extensive exploration of its synthesis and biological functions. Recently, Wang and coauthors disclosed the first successfully total synthesis of hoiamide C, which was accomplished by using a highly efficient convergent approach [111].

Hoiamide A shows modest cytotoxicity against H460 human lung adenocarcinoma and neuro-2a mouse neuroblastoma cells with IC_50_ values of 11.2 μM and 2.1 μM, respectively [109]. Hoiamide B displays low cytotoxicity against H460 with an IC_50_ value of 8.3 μM, but shows no inhibition of neuro-2a neuroblastoma cells, indicating that replacing the Hiva residue in hiamide A by a Hmpa residue in hiamide B remarkably decreases cytotoxicity for neuro-2a [109]. Moreover, the linear analog hoiamide C shows no significant inhibitory activity for either H460 or neuro-2a cell line, suggesting that the macrocyclic ring and the hydrogen bond donors at C-3, C-13, and C-37 positions in 1 and 2 are important structural features for the bioactivity of hoiamides [109].

Among hoiamides A–D, hoiamide A and B are site 2 activators of VGSCs. Hoiamide A produces a rapid and concentration-dependent elevation of [Na^+^]_i_ with an EC_50_ value of 2.31 μM in neocortical neurons and TTX antagonizes hoiamide A-induced [Na^+^]_i_ elevation [108]. In comparison, BTX, another site 2 agonist of VGSCs, causes a rapid and concentration-dependent elevation of neuronal [Na^+^]_i_ with an EC_50_ value of 11.4 nM. BTX induce the maximum elevation of [Na^+^]_i_ at concentrations greater than 60 mM, whereas the maximal response for hoiamide A was less than 20 mM [108]. Moreover, hoiamide A inhibits the specific binding of [3H]BTX A and 20-α-benzoate ([3H]BTX) to neurotoxin site 2 on VGSCs in neocortical neurons, providing direct evidence that hoiamide A is an agonist of VGSCs through interacting with the neurotoxin site 2 [108]. In addition, deltamethrin, an agonist binding to a site distinct from sites 1–6 and allosterically coupled to sites 2, 3, and 5, enhances hoiamide A-induced elevation of [Na^+^]_i_ in neocortical neurons, which is in agreement with the binding of hoiamide A to neurotoxin site 2 [108]. Similar to hoiamide A, hoiamide B stimulates Na^+^ influx with an EC_50_ value of 3.9 µM in mouse neocortical neurons. Considering the structural similarity between hoiamide A and B, it is plausible to speculate that hoiamide B is also a site 2 activator of VGSCs [109].

### 2.8. Palmyrolide A

**Figure 8:**
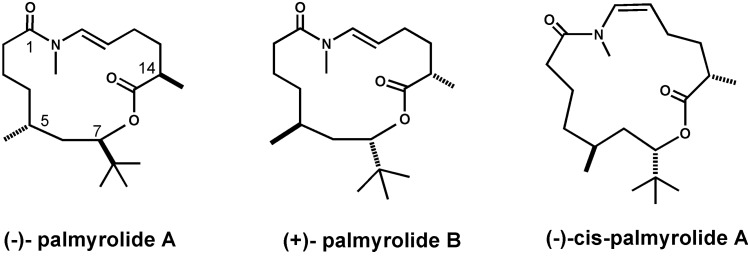
Structures of palmyrolide A [112].

Palmyrolide A (C_20_H_36_O_3_N, *m/z*: 338.2690) (Figure 8) is a novel neuroactive macrolide that features a rare *N*-methyl enamide and *t*-butyl branch [112]. In 2010, Gerwick and co-workers reported the isolation, structure determination, and biological activity of palmyrolide A. It was isolated from a cyanobacterial assemblage of *Leptolyngbya* cf and *Oscillatoria* spp. collected in the Northern Pacific at Palmyra Atoll [113]. Palmyrolide A displays interesting bioactivities including VGSC blockage and Ca^2+^ oscillation inhibition [113]. Its unique structure has attracted attentions of many researchers. In 2012, Maio and coworkers reported the first total synthesis of (+)-ent-palmyrolide A and the first asymmetric total synthesis, along with the absolute configurations of products they obtained [114,115]. Meanwhile, Brimble and coworkers accomplished a total synthesis of the initially reported and the revised structures [116]. In 2013, Reddy *et al.* disclosed the shortest synthetic route for (+)-palmyrolide A and produced (−)-*cis*-palmyrolide A for the first time by modifying Maio’s macrocyclization conditions [112].

Gerwick and coauthors demonstrated that palmyrolide A acts on VGSCs in neuro-2a cells [113]. Palmyrolide A suppresses veratridine- and ouabain-induced Na^+^ overload and shows cytotoxicity with an IC_50_ value of 5.2 µM [113]. Moreover, palmyrolide A significantly inhibits Ca^2+^ oscillations in murine cerebrocortical neurons with an average IC_50_ value of 3.7 µM (2.29–5.98 µM, 95% CI), although it is not cytotoxic when tested against H-460 human lung adenocarcinoma cells up to 20 µM [113]. These results indicate that palmyrolide A may be a suitable probe for pharmacological study of VGSCs.

### 2.9. Palmyramide A

**Figure 9:**
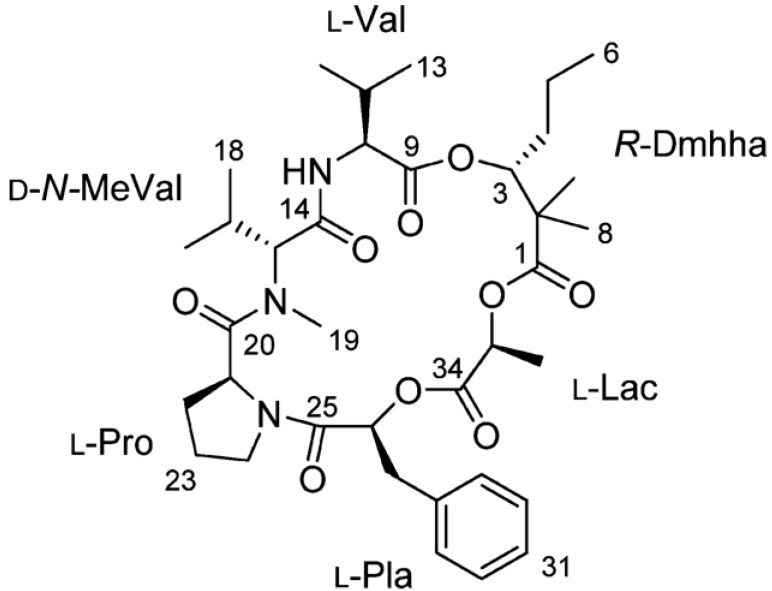
Structures of palmyramide A [117].

Palmyramide A (C_36_H_53_N_3_O_9_, *m/z*: 672.3852) (Figure 9), a novel cyclic depsipeptide, was purified by Gerwick *et al.* in 2010 from a consortium of cyanobacteria and red algae collected at Palmyra Atoll [117]. The cyclic depsipeptide is composed of three amino acids and three hydroxyl acids in an unusual arrangement. Among the three hydroxyl acids, 2,2-dimethyl-3-hydroxyhexanoic acid (Dmhha) is the most unusual which has only been reported previously in guineamides E and F [118]. The planar structure of palmyramide A was determined by 1D and 2D NMR studies and mass spectrometry.

Palmyramide A is a VGSC blocker. It inhibits veratridine- and ouabain-induced Na^+^ overload in cerebrocortical neurons with an IC_50_ value of 17.2 µM. In addition, palmyramide A displays modest cytotoxic activities against cancer cells, inhibiting H-460 human lung carcinoma cells with an IC_50_ value of 39.7 µM [117].

## 3. Pharmaceutical Potential of Shellfish Toxins

The past two decades witnessed a boom in reports of secondary metabolites purified from marine and freshwater algae, among which shellfish toxins have drawn considerable attention for their structural diversity and biological activities. Particularly, the activities of shellfish toxins on VGSCs have attracted significant interest in biomedical research to explore the therapeutic potential of these compounds. Selective ion channel blockers have proved to be effective analgesics. Ziconotide (trade name Prialt^®^), a synthetic version of ω-conotoxin MVIIA, which selectively blocks N-type voltage-gated Ca^2+^ channels, reduces chronic pain in spinal cord injury and became the first marine natural product approved for pain management by the US Food and Drug Administration in 2004 [119,120,121,122]. Advances in biomedical research in recent years have demonstrated that VGSCs are involved in pain management. Loss-of-function mutations of Nav1.7 cause a complete absence of pain sensation in patients [123,124] while gain-of-function mutations lead to painful neuropathies [125,126]. In 2008, Lewis *et al.* demonstrated that MrVIB (μO-conotoxin) produced significant analgesic effects in neuropathic and inflammatory pain by selectively blocking Nav1.8, a VGSC specifically expressed in sensory neurons [127]. Shellfish toxins may represent a novel class of analgesics through regulation of VGSCs activity. We hereby discuss the pharmacological potential of shellfish toxins with a focus on STX and its analogs.

STX and its analogs selectively target VGSCs and block Na^+^ currents via binding to site 1 with nanomolar affinity, leading to inhibition of nerve impulses, which may result in paralytic shellfish poisoning. Meanwhile, they also have significant potential as extraordinary anesthetics and analgesics. The therapeutic potential of STX as a highly selective and long lasting blocker of nerve conduction was recognized as early as 1975. However, its innate systematic toxicity has hampered its clinical development. Recent advances in drug delivery techniques may provide new strategies to facilitate the clinical development of STX. Recently, Kohane reported liposomal formulations for a mixture of STX, bupivacaine, and dexamethasone, which delivered prolonged duration of local anesthesia with minimal systemic toxicity [128]. The formulated mixture produced sciatic nerve blockade lasting up to 7.5 days in male Sprague–Dawley rats. Systemic toxicity was only observed with high loadings of dexamethasone, which caused increased release of liposomal STX. Mild myotoxicity occurred only in formulations containing bupivacaine. There was no nerve injury on Epon-embedded sections or up-regulation of the four genes associated with nerve injury in the dorsal root ganglia. The authors thus concluded that controlled release of STX and similar compounds is capable of providing prolonged nerve blockage with minimal systemic and local toxicity [128].

Gonyautoxins display remarkable therapeutic effects in treatment of pain caused by anal fissures. In 2004, Garrido *et al.* reported that gonyautoxin 2/3 epimers effectively reduced anal tone when injected into anal sphincter of healthy adults [129]. In this study, gonyautoxin 2/3 epimers were administered by local infiltration in the anal internal sphincter in healthy adult volunteers, and the beneficial effects were evaluated by anoscopy, electromyography, and anorectal manometry before and after toxin application. Gonyautoxin administration significantly decreased anal maximal voluntary contraction pressure, produced immediate relaxation, and brought statistically decrease in anal tone (*P* < 0.001). Later, Garrido *et al.* reported effective treatment of chronic anal fissure with repeated gonyautoxin injection. Gonyautoxin 100 units was intrasphincterically injected and infiltrated to 23 chronic anal fissure patients every four days. Anorectal pressure was recorded before and four minutes after each infiltration. Results showed that total remissions were achieved within 7–14 days with an average of 8.2 ± 2.4 days. Importantly, no relapses or side effects were observed during a 10-month follow up study [43]. Currently gonyautoxin injections are being intensively exploited as a new therapy for pain management in anal fissure patients [130]. In addition, gonyautoxin by local infiltration has been shown to be a safe and effective treatment of chronic tension-type headache [44].

Taken together, shellfish toxins such as STX and gonyautoxins may provide a new rich source for development of novel pain therapies. In the past ten years, significant progress has been made in biosynthetic route elaboration and total synthesis of cyanobacterial toxins, such as STX. With more and more new members being identified, shellfish toxins may provide great opportunity for pharmaceutical discovery and development.

## 4. Conclusions

Shellfish toxins exhibit novel and diverse structural features, intriguing biological activities, and significant pharmaceutical potential, which place them in the center of current studies of marine natural products. Systematic and comprehensive investigation of structure–activity relationships of shellfish toxins enables the design and synthesis of toxins with improved properties. Shellfish toxins with desirable VGSC subtype selectivity combined with suitable delivery vehicles may deliver superior therapeutic benefits with minimal toxicity.

## References

[1] Cestèle S., Catterall W.A. (2000). Molecular mechanisms of neurotoxin action on voltage-gated sodium channels. Biochimie.

[2] Ogata N., Ohishi Y. (2002). Molecular diversity of structure and function of the voltage-gated Na^+^ channels. Jpn. J. Pharmacol..

[3] Isom L.L., DeJongh K.S., Patton D.E., Reber B.F., Offord X.J., Charbonneau H., Walsh K., Goldin A.L., Catterall W.A. (1992). Primary structure and functional expression of the β_1_ Subunit of the rat brain sodium channel. Science.

[4] Isom L.L., Ragsdale D.S., De Jongh K.S., Westenbroek R.E., Reber B.F., Scheuer X.T., Catterall W.A. (1995). Structure and function of the β_2_ subunit of brain sodium channels, a transmembrane glycoprotein with a CAM motif. Cell.

[5] Morgan K., Stevens E.B., Shah B., Cox P.J., Dixon A.K., Lee K., Pinnock R.D., Hughes J., Richardson P.J., Mizuguchi K., Jackson A.P. (2000). β_3_: An additional auxiliary subunit of the voltage-sensitive sodium channel that modulates channel gating with distinct kinetics. Proc. Natl. Acad. Sci. USA.

[6] Aman T.K., Grieco-Calub T.M., Chen C., Rusconi R., Slat E.A., Isom L.L., Raman I.M. (2009). Regulation of persistent Na current by interactions between β subunits of voltage-gated Na channels. J. Neurosci..

[7] Patino G.A., Isom L.L. (2010). Electrophysiology and beyond: multiple roles of Na^+^ channel β subunits in development and disease. Neurosci. Lett..

[8] Wang J., Yarov-Yarovoy V., Kahn R., Gordon D., Gurevitz M., Scheuer M., Catterall W.A. (2011). Mapping the receptor site for α-scorpion toxins on a Na^+^ channel voltage sensor. Proc. Natl. Acad. Sci. USA.

[9] Klint J.K., Senff S., Rupasinghe D.B., Er S.Y., Herzig V., Nicholson G.M., King G.F. (2012). Spider-venom peptides that target voltage-gated sodium channels: pharmacological tools and potential therapeutic leads. Toxicon.

[10] Catterall W.A., Cestèle S., Yarov-Yarovoy V., Yu F.H., Konoki K., Scheuer T. (2007). Voltage-gated ion channels and gating modifier toxins. Toxicon.

[11] King G.F., Escoubas P., Nicholson G.M. (2008). Peptide toxins that selectively target insect Na_V_ and Ca_V_ channels. Channels.

[12] Catterall W.A. (2010). Ion channel voltage sensors: structure, function, and pathophysiology. Neuron.

[13] Smith M.R., Goldin A.L. (1997). Interaction between the sodium channel inactivation linker and domain III S4-S5. Biophys. J..

[14] Catterall W.A. (2000). From ionic currents to molecular mechanisms: The structure and function of voltage-gated sodium channels. Neuron.

[15] Brackenbury W.J., Isom L.L. (2008). Voltage-gated Na^+^ channels: Potential for β subunits as therapeutic targets. Expert Opin. Ther. Targets.

[16] Mantegazza M., Curia G., Biagini G., Ragsdale D.S., Avoli M. (2010). Voltage-gated sodium channels as therapeutic targets in epilepsy and other neurological disorders. Lancet Neurol..

[17] England S., de Groot M.J. (2009). Subtype-selective targeting of voltage-gated sodium channels. Br. J. Pharmacol..

[18] Catterall W.A., Goldin A.L., Waxman S.G. (2005). International Union of Pharmacology. XLVII. Nomenclature and structure-function relationships of voltage-gated sodium channels. Pharmacol. Rev..

[19] Ogta N., Tatebayashi H. (1993). Kinetic analysis of two types of Na^+^ channels in rat dorsal root ganglia. J. Physiol..

[20] Goldin A.L. (2001). Resurgence of sodium research. Annu. Rev. Physiol..

[21] Fainzilber M., Kofman O., Zlotkin E., Gordon D. (1994). A new neurotoxin receptor site on sodium channels is identified by a conotoxin that affects sodium channel inactivation in molluscs and acts as an antagonist in rat brain. J. Biol. Chem..

[22] Lefebvre K.A., Bill B.D., Erickson A., Baugh K.A., O’Rourke L., Costa P.R., Nance S., Trainer V.L. (2008). Characterization of intracellular and extracellular saxitoxin levels in both field and cultured *Alexandrium* spp. samples from Sequim Bay, Washington. Mar. Drugs.

[23] Usup G., Kulis D.M., Anderson D.M. (1994). Growth and toxin production of the toxic dinoflasellate *Pyodinium bahamense* var. *compressum* in laboratory cultures. Nat. Toxins.

[24] Oshima Y., Blackburn S.I., Hallegraeff G.M. (1993). Comparative study on paralytic shellfish toxin of the dinoflagellate *Gymnodinium catenatum* from three different countries profiles. Mar. Biol..

[25] Daugbjerg N., Hansen G., Larsen J., Mosestrup Ø. (2000). Phylogeny of some of the major genera of dinoflagellates based on ultrastructure and partial LSU rDNA sequence data, including the erection of three new genera of unarmoured dinoflagellates. Phycologia.

[26] Smith F.M., Wood S.A., Ginkel R.V., Broady P.A., Gaw S. (2011). First report of saxitoxin production by a species of the freshwater benthic cyanobacterium, *Scytonema agardh*. Toxicon.

[27] Wang D.Z. (2008). Neurotoxins from marine dinoflagellates: A brief review. Mar. Drugs.

[28] Cusick K.D., Sayler G.S. (2013). An overview on the marine neurotoxin, saxitoxin: Genetics, molecular targets, methods of detection and ecological functions. Mar. Drugs.

[29] Watkins S.M., Reich A., Fleming L.E., Hammond R. (2008). Neurotoxic shellfish poisoning. Mar. Drugs.

[30] Van Dolah F.M. (2000). Marine algal toxins: Origins, health effects, and their increased occurrence. Environ. Health Perspect..

[31] Anderson D.M., Glibert P.M., Burkholder J.M. (2002). Harmful algal blooms and eutrophication: Nutrient sources, composition, and consequences. Estuaries.

[32] Sellner K.G., Doucette G.J., Kirkpatrick G.J. (2003). Harmful algal blooms: causes, impacts and detection. J. Ind. Microbiol. Biotechnol..

[33] Rogart R.B. (1986). High-STX-affinity *vs.* low-STX-affinity Na^+^ channel subtypes in nerve, heart, and skeletal muscle. Ann. N. Y. Acad. Sci..

[34] Wang J., Salata J.J., Bennett P.B. (2003). Saxitoxin is a gating modifier of hERG K^+^ channels. J. Gen. Physiol..

[35] Su Z., Sheets M., Ishida H., Li F., Barry W.H. (2004). Saxitoxin blocks L-type I_Ca_. J. Pharmacol. Exp. Ther..

[36] Böttinger H., Béress L., Habermann E. (1986). Involvement of (Na^+^ + K^+^)-ATPase in binding and actions of palytoxin on human erythrocytes. Biochim. Biophys. Acta Biomembr..

[37] Thomas P., Stephens M., Wilkie G., Amar M., Lunt G.G., Whiting P., Gallagher T., Pereira E., Alkondon M., Albuquerque E.X., Wonnacott S. (1993). (+)-Anatoxin-a is a potent agonist at neuronal nicotinic acetylcholine receptors. J. Neurochem..

[38] Wiese M., D’Agostino P.M., Mihali T.K., Moffitt M.C., Neilan B.A. (2010). Neurotoxic alkaloids: Saxitoxin and its analogs. Mar. Drugs.

[39] Schantz E.J., Mold J.D., Stanger D.W., Shavel J., Riel F.J., Bowden J.P., Lynch J.M., Wyler R.S., Riecel B., Sommer H. (1957). Paralytic shellfish poison. VI. A procedure for the isolation and purification of the poison from toxic clam and mussel tissues. J. Am. Chem. Soc..

[40] Terlau H., Heinemann S.H., Stühmer W., Pusch M., Conti F., Imoto K., Numa S. (1991). Mapping the site of block by tetrodotoxin and saxitoxin of sodium channel II. FEBS Lett..

[41] Noda M., Suzuki H., Numa S., Stiihmer W. (1989). A single point mutation confers tetrodotoxin and saxitoxin insensitivity on the sodium channel II. FEBS Lett..

[42] Barnet C.S., Tse J.Y., Kohane D.S. (2004). Site 1 sodium channel blockers prolong the duration of sciatic nerve blockade from tricyclic antidepressants. Pain.

[43] Garrido R., Lagos N., Lagos M., Rodríguez-Navarro A.J., Garcia C., Truan D., Henriquez A. (2007). Treatment of chronic anal fissure by gonyautoxin. Colorectal Dis..

[44] Lattes K., Venegas P., Lagos N., Lagos M., Pedraza L., Rodriguez-Navarro A.J., García C. (2009). Local infiltration of gonyautoxin is safe and effective in treatment of chronic tension-type headache. Neurol. Res..

[45] Negri A.P., Jones G.J., Hindmarsh M. (1995). Sheep mortality associated with paralytic shellfish poisons from the cyanbacterium *Anabaena circinalis*. Toxicon.

[46] Reyero M., Cacho E., Martínez A., Vázquez J., Marina A,, Fraga S., Franco J.M. (1999). Evidence of saxitoxin derivatives as causative agents in the 1997 mass mortality of monk seals in the Cape Blanc Peninsula. Nat. Toxins.

[47] Anderson D.M. (1994). Red tides. Sci. Am..

[48] Stewart I., Seawright A.A., Shaw G.R. (2008). Cyanobacterial poisoning in livestock, wild mammals and birds—an overview. Cyanobacterial Harmful Algal Blooms: State of the Science and Research Needs.

[49] Trainer V.L., Baden D.G. (1999). High affinity binding of red tide neurotoxins to marine mammal brain. Aquat. Toxicol..

[50] Halsetead B.W. (1978). Poisonous and Venomous Marine Animals of the World.

[51] Llewellyn L.E. (2006). Saxitoxin, a toxic marine natural product that targets a multitude of receptors. Nat. Prod. Rep..

[52] Pearson L., Mihali T., Moffitt M., Kellmann R., Neilan B. (2010). On the chemistry, toxicology and genetics of the cyanobacterial toxins, microcystin, nodularin, saxitoxin and cylindrospermopsin. Mar. Drugs.

[53] Lagos N., Andrinolo D., Botana L.M. (2000). Paralytic shellfish poisoning (PSP): Toxicology and kinetics. Seafood and Freshwater Toxins: Pharmacology, Physiology and Detection.

[54] Kao C.Y., Falconer I.R. (1993). Paralytic shellfish poisoning. Algal Toxins in Seafood and Drinking Water.

[55] Hall S., Strichartz G., Moczydlowski E., Ravindran A., Reichardt P.B., Hall S., Reichardt P.B. (1990). The saxitoxins: Sources, chemistry and pharmacology. Marine Toxins. Origin, Structure and Pharmacology.

[56] Schantz E.J., Ghazarossian V.E., Schnoes H.K., Strong F.M., Springer J.P., Pezzanite J.O., Clardy J. (1975). The structure of saxitoxin 1. J. Am. Chem. Soc..

[57] Bordner J., Thiessen W.E., Bates H.A., Rapoport H. (1975). The structure of a crystalline derivative of saxitoxin. The structure of saxitoxin. J. Am. Chem. Soc..

[58] Rogers S.R., Rapport H. (1980). The p*K*_a_’s of saxitoxin. J. Am. Chem. Soc..

[59] Shimizu Y., Hsu C.P., Genenah A. (1981). Structure of saxitoxin in solutions and stereochemistry of dihydrosaxitoxins. J. Am. Chem. Soc..

[60] Schantz E.J., Lynch J.M., Vayvada G., Matsumoto K., Rapoport H. (1966). The purification and characterization of the poison produced by *Gonyaulax catenella* in axenic culture. Biochemistry.

[61] Strichartz G. (1984). Structural determinants of the affinity of saxitoxin for neuronal sodium Channels. Electrophysiological studies on frog peripheral nerve. J. Gen. Physiol..

[62] Van Dolah F.M., Botana L. (2000). Diversity of Marine and Freshwater Algal Toxins. Seafood Toxicology: Pharmacology, Physiology and Detection.

[63] Hille B. (1975). The receptor for tetrodotoxin and saxitoxin. A structural hypothesis. Biophys. J..

[64] Kao C.Y., Walker S.E. (1982). Active groups of saxitoxin and tetrodotoxin as deduced from actions of saxitoxin analogues on frog muscle and squid axon. J. Physiol..

[65] Shiinizu Y. (1982). Recent progress in marine toxin research. Pure Appl. Chem..

[66] Numa S., Noda M. (1986). Molecular structure of sodium channels. Ann. N. Y. Acad. Sci..

[67] Hartshorne R.P., Catterall W.A. (1984). The sodium channel from rat brain. Purification and subunit composition. J. Biol. Chem..

[68] Walker J.R., Novick P.A., Parsons W.H., McGregor M., Zablocki J., Pande V.S., Du Bois J. (2012). Marked difference in saxitoxin and tetrodotoxin affinity for the human nociceptive voltage-gated sodium channel (Nav1.7). Proc. Natl. Acad. Sci. USA.

[69] Steidinger K.A. (1973). Phytoplankton ecology: A conceptual review based on eastern. Gulf of Mexico research. Crit. Rev. Microbiol..

[70] Baden D.G. (1983). Marine food-borne dinoflagellate toxins. Int. Rev. Cytol..

[71] Baden D.G., Mende T.J. (1982). Toxicity of two toxins from the Florida red tide marine dinoflagellate, *Ptychodiscus brevis*. Toxicon.

[72] Poli M., Mende T.J., Baden D.G. (1986). Brevetoxins, unique activators of voltage-sensitive sodium channels bind to specific sites in rat brain synaptosomes. Mol. Pharmacol..

[73] Baden D., Fleming L.E., Bean J.A., deWolf F.A (1995). Marine Toxins. Handbook of Clinical Neurology: Intoxications of the Nervous System Part H. Natural Toxins and Drugs.

[74] Landsberg J.H. (2002). The effects of harmful algal blooms on aquatic organisms. Rev. Fish. Sci..

[75] Flewelling L.J., Naar J.P., Abbott J.P., Baden D.G., Barros N.B., Bossart G.D., Bottein M.Y., Hammond D.G., Haubold E.M., Heil C.A. (2005). Brevetoxicosis: Red tides and marine mammal mortalities. Nature.

[76] Sagir Ahmed M.D., Arakawa O., Onoue Y., Lassus P., Arzul G., Erhard E., Gentien P., Marcaillou C. (1995). Toxicity of cultured *Chatonella marina*. Harmful Marine Algal Blooms.

[77] Khan S., Arakawa O., Onoue Y. (1997). Neurotoxins in a toxic red tide of *Heterosigma akashiwo* (Raphidophyceae) in Kagoshima Bay, Japan. Aquac. Res..

[78] Hallegraeff G.M., Munday B.L., Baden D.G., Whitney P.L., Reguera B., Blanco J., Ferandz M.L., Wyatt T. (1998). *Chatonnella maria* raphidophyte bloom associated with mortality of cultured bluefin tuna (*Thunnus maccoyii*) in south Australia. Harmful Algae.

[79] Morris P.D., Campbell D.S., Taylor T.J., Freeman J.I. (1991). Clinical and epidemiological features of neurotoxic shellfish poisoning in North Carolina. Am. J. Public Health.

[80] Kirkpatrick B., Fleming L.E., Squicciarini D., Backer L.C., Clark R., Abraham W., Benson J., Chenge Y.S., Johnson D., Pierce R. (2004). Literature review of Florida red tide: Implications for human health effects. Harmful Algae.

[81] Gallagher J.P., Shinnick-Gallaqher P. (1980). Effect of *Gymnodinium breve* toxin in the rat phrenic nerve diaphragm preparation. Br. J. Pharmacol..

[82] Trainer V.L., Thomsen W.J., Catterall W.A., Baden D.G. (1991). Photoaffinity labeling of the brevetoxin receptor on sodium channels in rat brain synaptosomes. Mol. Pharmacol..

[83] Rein K.S., Baden D.G., Gawley R.E. (1994). Conformational analysis of the sodium channel modulator, brevetoxin A, comparison with brevetoxin B conformations, and a hypothesis about the common pharmacophore of the “site 5” toxins. J. Org. Chem..

[84] Baden D.G., Bourdelais A.J., Jacocks H., Michelliza S., Naar J. (2005). Natural and derivative brevetoxins: Historical background, multiplicity, and effects. Environ. Health Perspect..

[85] Orjala J., Nagle D.G., Hsu V.L., Gerwick W.H. (1995). Antillatoxin: An exceptionally ichthyotoxic cyclic lipopeptide from the tropical cyanobacterium *Lyngbya majuscula*. J. Am. Chem. Soc..

[86] Yokokawa F., Fujiwara H., Shioiri T. (2000). Total synthesis and revision of absolute stereochemistry of antillatoxin, an ichthyotoxic cyclic lipopeptide from marine cyanobacterium *Lyngbya majuscula*. Tetrahedron.

[87] Li W.I., Marquez B.L., Okino T., Yokokawa F., Shioiri T., Gerwick W.H., Murray T.F. (2004). Characterization of the preferred stereochemistry for the neuropharmacologic actions of antillatoxin. J. Nat. Prod..

[88] Osborne N.J., Webb P.M., Shaw G.R. (2001). The toxins of *Lyngbya majuscula* and their human and ecological health effects. Environ. Int..

[89] Nogle L.M., Okino T., Gerwick W.H. (2001). Antillatoxin B, a neurotoxic lipopeptide from the marine cyanobacterium lyngbya majuscule. J. Nat. Prod..

[90] Wu M., Okino T., Nogle L.M., Marquez B.L., Williamson R.T., Sitachitta N., Berman F.W., Murray T.F., McGough K., Jacobs R. (2000). Structure, synthesis, and biological properties of kalkitoxin, a novel neurotoxin from the marine cyanobacterium *Lyngbya majuscula*. J. Am. Chem. Soc..

[91] Aráoz R., Molgó J., Tandeau de Marsac N. (2010). Neurotoxic cyanobacterial toxins. Toxicon.

[92] Yokokawa F., Fujiwara H., Shioiri T. (1999). Total synthesis and revision of absolute configuration of antillatoxin, an ichthyotoxic cyclic lipopeptide of marine origin. Tetrahedron Lett..

[93] Yokokawa F., Shioiri T. (1998). Total synthesis of antillatoxin, an ichthyotoxic cyclic lipopeptide, having the proposed structure. What is the real structure of antillatoxin?. J. Org. Chem..

[94] Berman F.W., Gerwick W.H., Murray T.F. (1999). Antillatoxin and kalkitoxin, ichthyotoxins from the tropical cyanobacterium *Lyngbya majuscula*, induce distinct temporal patterns of NMDA receptor-mediated neurotoxicity. Toxicon.

[95] Li W.I., Berman F.W., Okino T., Yokokawa F., Shioiri T., Gerwick W.H., Murray T.F. (2001). Antillatoxin is a marine cyanobacterial toxin that potently activates voltage-gated sodium channels. Proc. Natl. Acad. Sci. USA.

[96] Cao Z., George J., Gerwick W.H., Baden D.G., Rainier J.D., Murray T.F. (2008). Influence of lipid-soluble gating modifier toxins on sodium influx in neocortical neurons. J. Pharmacol. Exp. Ther..

[97] Jabba S.V., Prakash A., Dravid S.M., Gerwick W.H., Murray T.F. (2010). Antillatoxin, a novel lipopeptide, enhances eeurite outgrowth in immature cerebrocortical neurons through activation of voltage-gated sodium channels. J. Pharmacol. Exp. Ther..

[98] Cao Z., Gerwick W.H., Murray T.F. (2010). Antillatoxin is a sodium channel activator that displays unique efficacy in heterologously expressed rNa_v_1.2, rNa_v_1.4 and rNa_v_1.5 alpha subunits. BMC Neurosci..

[99] Nogle L.M., Gerwick W.H. (2003). Diverse secondary metabolites from a Puerto Rican collection of *Lyngbya majuscula*. J. Nat. Prod..

[100] White J.D., Lee C.S., Xu Q. (2003). Total synthesis of (+)-kalkitoxin. Chem. Commun. (Camb.).

[101] White J.D., Xu Q., Lee C.S., Valeriote F.A. (2004). Total synthesis and biological evaluation of (+)-kalkitoxin, a cytotoxic metabolite of the cyanobacterium *Lyngbya majuscule*. Org. Biomol. Chem..

[102] Umezawa T., Sueda M., Kamura T., Kawahara T., Han X., Okino T., Matsuda F. (2012). Synthesis and biological activity of kalkitoxin and its analogues. J. Org. Chem..

[103] LePage K.T., Goeger D., Yokokawa F., Asano T., Shioiri T., Gerwick W.H., Murray T.F. (2005). The neurotoxic lipopeptide kalkitoxin interacts with voltage-sensitive sodium channels in cerebellar granule neurons. Toxicol. Lett..

[104] Edwards D.J., Marquez B.L., Nogle L.M., McPhail K., Goeger D.E., Roberts M.A., Gerwick W.H. (2004). Structure and biosynthesis of the jamaicamides, new mixed polyketide-peptide neurotoxins from the marine cyanobacterium *Lyngbya majuscule*. Chem. Biol..

[105] Graf K.M., Tabor M.G., Brown M.L., Paige M. (2009). Synthesis of (*s*)-jamaicamide C carboxylic acid. Org. Lett..

[106] Manger R.L., Leja L.S., Lee S.Y., Hungerford J.M., Hokama Y., Dickey R.W., Granade H.R., Lewis R., Yasumoto T., Wekell M.M. (1995). Detection of sodium channel toxins: Directed cytotoxicity assays of purified ciguatoxins, brevetoxins, saxitoxins, and seafood extracts. J. AOAC Int..

[107] Choi H., Engene N., Smith J.E., Preskitt L.B., Gerwick W.H. (2010). Crossbyanols A–D, toxic brominated polyphenyl ethers from the Hawai’ian bloom-forming cyanobacterium *Leptolyngbya crossbyana*. J. Nat. Prod..

[108] Pereira A., Cao Z., Murray T.F., Gerwick W.H. (2009). Hoiamide A, a sodium channel activator of unusual architecture from a consortium of two papua new Guinea Cyanobacteria. Chem. Biol..

[109] Choi H., Pereira A.R., Cao Z., Shuman C.F., Engene N., Byrum T., Matainaho T., Murray T.F., Mangoni A., Gerwick W.H. (2010). The hoiamides, structurally intriguing neurotoxic lipopeptides from Papua New Guinea marine cyanobacteria. J. Nat. Prod..

[110] Malloy K.L., Choi H., Fiorilla C., Valeriote F.A., Matainaho T., Gerwick W.H. (2012). Hoiamide D, a marine cyanobacteria-derived inhibitor of p53/MDM2 interaction. Bioorg. Med. Chem. Lett..

[111] Wang L., Xu Z., Ye T. (2011). Total synthesis of hoiamide C. Org. Lett..

[112] Philkhana S.C., Seetharamsingh B., Dangat Y.B., Vanka K., Reddy D.S. (2013). Synthesis of palmyrolide A and its *cis*-isomer and mechanistic insight into *trans–cis* isomerisation of the enamide macrocycle. Chem. Commun..

[113] Pereira A.R., Cao Z., Engene N., Soria-Mercado I.E., Murray T.F., Gerwick W.H. (2010). Palmyrolide A, an unusually stabilized neuroactive macrolide from palmyra atoll cyanobacteria. Org. Lett..

[114] Tello-Aburto R., Newar T.D., Maio W.A. (2012). Evolution of a protecting-group-free total synthesis: Studies en route to the neuroactive marine macrolide (−)-palmyrolide A. J. Org. Chem..

[115] Tello-Aburto R., Johnson E.M., Valdez C.K., Maio W.A. (2012). Asymmetric total synthesis and absolute stereochemistry of the neuroactive marine macrolide palmyrolide A. Org Lett..

[116] Wadsworth A.D., Furkert D.P., Sperry J., Brimble M.A. (2012). Total synthesis of the initially reported and revised structures of the neuroprotective agent palmyrolide A. Org. Lett..

[117] Taniguchi M., Nunnery J.K., Engene N., Esquenazi E., Byrum T., Dorrestein P.C., Gerwick W.H. (2010). Palmyramide A, a cyclic depsipeptide from a palmyra atoll collection of the marine cyanobacterium *Lyngbya majuscule*. J. Nat. Prod..

[118] Tan L.T., Sitachitta N., Gerwick W.H. (2003). The guineamides, novel cyclic depsipeptides from a Papua New Guinea collection of the marine cyanobacterium *Lyngbya majuscule*. J. Nat. Prod..

[119] Glaser K.B., Mayer A.M.S. (2009). A renaissance in marine pharmacology: from preclinical curiosity to clinical reality. Biochem. Pharmacol..

[120] Olivera B.M., Cruz L.J., de Santos V., LeCheminant G.W., Griffin D., Zeikus R., McIntosh J.M., Galyean R., Varga J., Gray W.R. (1987). Neuronal calcium channel antagonists. Discrimination between calcium channel cubtypes using ω-conotoxin from *Conus magus* venom. Biochemistry.

[121] Molinski T.F., Dalisay D.S., Lievens S.L., Saludes J.P. (2009). Drug development from marine natural products. Nat. Rev. Drug Discov..

[122] Klotz U. (2006). Ziconotide—a novel neuron-specific calcium channel blocker for the Intrathecal treatment of severe chronic pain—a short review. Int. J. Clin. Pharmacol. Ther..

[123] Goldberg Y.P., MacFarlane J., MacDonald M.L., Thompson J., Dube M.P., Mattice M., Fraser R., Young C., Hossain S., Pape T. (2007). Loss-of-function mutations in the Na_v_1.7 gene underlie congenital indifference to pain in multiple human populations. Clin. Genet..

[124] Cox J.J., Reimann F., Nicholas A.K., Thornton G., Roberts E., Springell K., Karbani G., Jafri H., Mannan J., Raashid Y. (2006). An *SCN9A* channelopathy causes congenital inability to experience pain. Nature.

[125] Fertleman C.R., Baker M.D., Parker K.A., Moffatt S., Elmslie F.V., Abrahamsen B., Ostman J., Klugbauer N., Wood J.N., Gardiner R.M. (2006). *SCN9A* mutations in paroxysmal extreme pain disorder: Allelic variants underlie distinct channel defects and phenotypes. Neuron.

[126] Han C., Rush A.M., Dib-Hajj S.D., Li S., Xu Z., Wang Y., Tyrrell L., Wang X., Yang Y., Waxman S.G. (2006). Sporadic onset of erythermalgia: A gain-of-function mutation in Na_v_1.7. Ann. Neurol..

[127] Ekberg J., Jayamanne A., Vaughan C.W., Aslan S., Thomas L., Mould J., Drinkwater R., Baker M.D., Abrahamsen B., Wood J.N. (2006). μO-conotoxin MrVIB selectively blocks Na_v_1.8 sensory neuron specific sodium channels and chronic pain behavior without motor deficits. Proc. Natl. Acad. Sci. USA.

[128] Epstein-Barash H., Shichor I., Kwon A.H., Hall S., Lawlor M.W., Langer R., Kohane D.S. (2009). Prolonged duration local anesthesia with minimal toxicity. Proc. Natl. Acad. Sci. USA.

[129] Garrido R., Laqos N., Lattes K., Azolas C.G., Bocic G., Cuneo A., Chiong H., Jensen C., Heríandez A., Fernández C. (2004). The Gonyautoxin 2/3 epimers reduces anal tone when injected in the anal sphincter of healthy adults. Biol. Res..

[130] Poh A., Tan K.Y., Seow-Choen F. (2010). Innovations in chronic anal fissure treatment: A systematic review. World J. Gastrointest. Surg..

